# Regulatory mechanism of *Scutellaria baicalensis* Georgi on bone cancer pain based on network pharmacology and experimental verification

**DOI:** 10.7717/peerj.14394

**Published:** 2022-11-17

**Authors:** Aitao Wang, Dongmei Guo, Hongyu Cheng, Hui Jiang, Xiaojuan Liu, Muer Tie

**Affiliations:** 1 Inner Mongolia People’s Hospital, Hohhot, China; 2 Inner Mongolia Medical University, Hohhot, China; 3 Baotou Medical College, Baotou, China

**Keywords:** Bone cancer pain, *Scutellaria baicalensis* Georgi, Network pharmacology, Targets, qRT-PCR

## Abstract

**Context:**

*Scutellaria baicalensis* Georgi (SBG) may relieve bone cancer pain (BCP) by regulating cell proliferation, angiogenesis, and apoptosis.

**Objective:**

The mechanism of SBG in the treatment of BCP remains to be further explored.

**Methods:**

The active compounds and targets of SBG were obtained from the Traditional Chinese Medicine Systems Pharmacology Database and Analysis Platform (TCMSP) and SwissTargetPrediction databases. BCP-related targets were screened from NCBI and GeneCards databases. Additionally, Cytoscape software was applied to construct network diagrams, and OmicShare platform was used to enrich Gene Ontology (GO) and pathways. Finally, the verification of active compounds and core targets was performed based on quantitative real-time PCR (qRT-PCR).

**Results:**

Interestingly, we identified baicalein and wogonin as the main active components of SBG. A total of 41 SBG targets, including VEGFA, IL6, MAPK3, JUN and TNF, were obtained in the treatment of BCP. In addition, pathways in cancer may be an essential way of SBG in the treatment of BCP. Experimental verification had shown that baicalein and wogonin were significantly related to BCP core targets.

**Conclusions:**

The active components of SBG have been clarified, and the mechanism of the active components in treating BCP has been predicted and verified, which provides an experimental and theoretical basis for the in-depth elucidation of the pharmacodynamics material basis and mechanism of SBG.

## Introduction

Common cancers, such as breast cancer and lung cancer, are prone to bone metastasis, which increase the morbidity, mortality, and more severe pain of patients (Hirai et al., 2019). It is clear that the pain caused by bone metastasis is a complex chronic disease, which can cause a persistent, severe breakthrough, neuropathic, and inflammatory pain, and seriously affects the quality of life and the independence of patients (Zajączkowska et al., 2019; Zhang et al., 2018). At present, the main treatment for bone cancer pain (BCP) is bisphosphonates, non-steroidal anti-inflammatory drugs, and opioids combined with radiotherapy or surgical intervention. It should be noted that zoledronic acid and pamidronate sodium have been approved by the U.S. Food and Drug Administration (FDA) (Buehlmann et al., 2019; Iranikhah et al., 2012). Nevertheless, long-term use of these therapies frequently produces serious side effects and cannot eliminate or relieve severe pain (Buehlmann et al., 2019). Traditional Chinese medicine (TCM) has few side effects relatively, which can relieve pain while improving immunity, thereby improving the therapeutic effect.

*Scutellaria baicalensis* Georgi (SBG), a bitter taste and cold nature, is the main herb of the famous prescription of Xiao Chai Hu Tang (Tao et al., 2017). Flavonoids are the main components of SBG, such as baicalein, skullcap flavone, wogonin, baicalin, wogonoside, oroxylin A, dihydrooroxylin A, and chrysin (Cui et al., 2015). Modern pharmacological studies have shown that SBG has anti-tumor, anti-apoptotic, analgesic, and anti-inflammatory activities. Currently, SBG has been used to treat many diseases, such as cancer, inflammation, and hypertension (Zhao et al., 2019b; Zhou et al., 2021). For the characteristic of having low side effects and significant biological activities, SBG has become an important source of screening drugs for the treatment of bone cancer pain (Gao et al., 2020; Sato et al., 2013). However, the mechanism of SBG in treating bone cancer pain is still unclear, and further research is needed.

**Figure 1:**
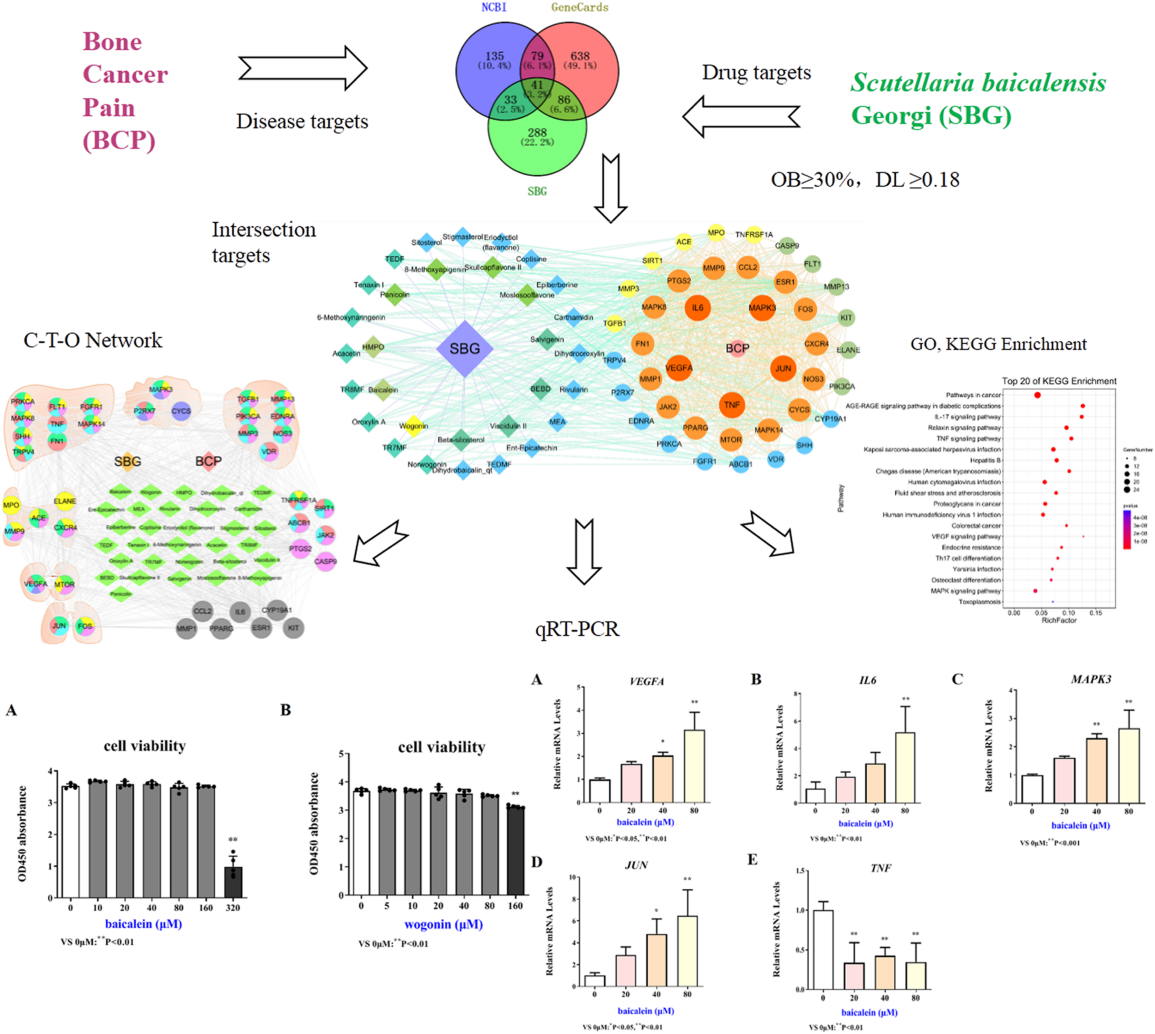
Technological road-map based on a cohesive integration strategy of network pharmacology and *in vitro* experiments. Compared with 0 µM, *P < 0.05, **P < 0.01.

Network pharmacology is a discipline that uses biological information technology to study the interaction between drugs and the body and its mechanisms, that is, from molecules, networks, and cells to tissues, organs and other levels to studying the mechanism of changes in body function caused by drugs in the treatment of diseases (Dai, Sun & Zhong, 2020; Zhang, Zhou & Ma, 2021). In this study, network pharmacology was used to explore the active components, key targets, and pathways of SBG in the treatment of BCP. Quantitative real-time PCR (qRT-PCR) were applied to verify the core components and targets, which provides a certain theoretical basis for the research on the mechanism of SBG in the treatment of BCP. The specific research flow chart was shown in Fig. 1.

## Materials and Methods

### Screening and target prediction of active ingredients of SBG

The Traditional Chinese Medicine Systems Pharmacology Database and Analysis Platform (TCMSP) was used to screen the components of SBG. In this study, the range of oral bioavailability (OB) greater than or equal to 30% and drug-likeness (DL) greater than or equal to 0.18 were used to screen active ingredients (Dai, Sun & Zhong, 2020; Wu et al., 2020). Subsequently, TCMSP and the SwissTargetPrediction databases (http://www.swisstargetprediction.ch/) (Yang et al., 2021) were applied to predict targets. Further, the standardization of targets were performed using the UniProt platform (https://www.uniprot.org/). The complex relationships between active compounds and their targets were visualized based on Cytoscape software (version 3.7.2).

### Collection of bone cancer pain targets

In order to reveal disease targets, the National Center for Biotechnology Information (NCBI, https://www.ncbi.nlm.nih.gov/) (Yang et al., 2021) and GeneCards (https://www.genecards.org/) (Dai, Sun & Zhong, 2020; Zhang, Zhou & Ma, 2021; Zhan et al., 2021) were used to retrieve the disease targets through searching the keywords “bone cancer pain”, “cancer bone pain”, “cancer bone metastasis”, “bone cancer”, and “cancer pain”. According to the targets score, the median was applied to screen the disease targets, and the duplicate values were deleted to obtain the core bone-cancer-pain-related targets.

### Intersection of active components and disease targets

To better investigate the complex relationship between components and diseases, the intersection targets of ingredients and diseases were obtained based on Venny 2.1.0 (http://bioinfogp.cnb.csic.es/tools/venny/). The STRING database (https://string-db.org/) was used to construct a protein-protein interaction network (PPI). Furthermore, R-3.6.3 software was used for the visualization of core targets, and Cytoscape 3.7.2 software was used to construct the component-target-disease (C-T-D) network diagram of the active ingredient and the intersection target.

### Gene Ontology (GO) term and Kyoto Encyclopedia of Genes and Genomes (KEGG) pathway enrichment analyses

In order to explore the biological information and signal pathways involved in the treatment of bone cancer pain by SBG (Wu et al., 2020), intersection targets were transformed into an “ensemble” form through the UniProt platform (http://www.UniProt.org/), and uploaded to the OmicShare platform (https://www.omicshare.com/) for GO and KEGG enrichment analyses and visualization.

### Organ distribution of targets

According to the BioGPS database (http://biogps.org/), the mRNA expression data of the intersection targets in different tissues can be harvested. In order to investigate the potential relationship of the active ingredients between SBG and BCP at the organ level. In this study, a triple value of the median mRNA expression was used to determine the tissue distribution of the targets. Similarly, targets without corresponding mRNA expression data were also excluded from the analysis. Finally, a component-target-organ (C-T-O) network was constructed through Cytoscape to more intuitively reflect the distribution of targets in the organization.

### Cells and culture conditions

BCP induces morphological and functional changes in brain structure, especially in hippocampal neuronal cellular plasticity (Wang et al., 2020). Currently, studies have found a decrease in the number of branches and dendritic length of CA3 pyramidal neurons in the hippocampus of mice with chronic sciatic nerve constriction injury (Tyrtyshnaia et al., 2019). Hippocampal neuron cell line (HT22) (CL-0595) was purchased from Wuhan Purcell Life Science Co., Ltd. (Wuhan, China). The HT22 cell line was cultured in Dulbecco’s modified Eagle’s medium (DMEM; Invitrogen, Camarillo, CA, USA) supplemented with 10% (v/v) fetal bovine serum (FBS; Gibco, Carlsbad, CA, USA), 100 µg/mL penicillin and 100 µg/mL streptomycin. Cells were grown at 37 °C in a humidified incubator with 5% carbon dioxide.

### Drug treatment of HT22 cells

Baicalein (purity ≧ 98%) and wogonin (purity ≧ 98%) were purchased from MedChemExpress (New Jersey, USA). The cytotoxicity control test of the drug was detected using Cell Counting Kit-8 (CCK-8) (Beyotime, Hangzhou, China). HT22 cells were seeded in a 96-well plate at a density of 3 × 10^5^, and after culturing for 24 h, different concentrations of baicalein (0, 10, 20, 40, 80, 160, 320 µM) and wogonin (0, 5, 10, 20, 40, 80, 160 µM) were added respectively. After 24 h of incubation, cell viability was detected with CCK8, and the OD value (450 nm) of each well was measured with a SpectraMAX M3 microplate reader (Molecular Devices, Sunnyvale, CA, USA), which was used to screen out the safe concentration range of the drug.

### QRT-PCR analysis

Total cellular RNA was extracted using Trizol reagent (Sigma, China) after 24 h incubation with various concentrations of baicalein (0, 20, 40, 80 µM) or wogonin (0, 10, 20, 40 µM). RNA purity and concentration were determined using a Nano Drop 2000 spectrophotometer (Thermo, USA). A reverse transcription kit (TaKaRa, Clontech) was applied to reverse transcribe RNA to cDNA. After reverse transcription, qRT-PCR was performed using the ABI 7500 Real-Time PCR System (Applied Biosystems, USA). The primer sequences were shown in Table 1, and the expression of VEGFA, IL-6, MAPK3, JUN and TNF target genes was detected. The samples were deformed at 95 °C for 30 s, then denatured at 95 °C for 40 cycles of 5 s and annealed at 60 °C for 34 s. Dissolution curve conditions were 95 °C for 15 s, 65 °C for 60 s, 95 °C for 30 s and 65 °C for 15 s. A system with a volume of 20 µL was used, 10 µL 2 × TB Green Premix Ex Taq II, 0.8 µL 10 µM forward primer and reverse primer, respectively, 0.4 µL 50 × ROX Reference Dye II, 2 µL cDNA, supplemented with double distilled water. GAPDH was used as the internal control, and the data were analyzed using the 2^−ΔΔCt^ method. The experiment was repeated three times.

**Table 1:** Primer sequences of qRT-PCR.

Gene names	Forward primer (5′–3′)	Reverse primer (3′–5′)
VEGFA	CTGCTGTAACGATGAAGCCCTG	GCTGTAGGAAGCTCATCTCTCC
IL6	TACCACTTCACAAGTCGGAGGC	CTGCAAGTGCATCATCGTTGTTC
MAPK3	CAACACCACCTGCGACCTTA	CCGGTTGGAGAGCATCTCAG
JUN	CAGTCCAGCAATGGGCACATCA	GGAAGCGTGTTCTGGCTATGCA
TNF	AGGCACTCCCCCAAAAGATG	CCACTTGGTGGTTTGTGAGTG

### Statistical analysis

The results of cell viability and target expression experiments were analyzed by one-way ANOVA. Data normalization was performed to control for the effect of changes in baseline parameters. GraphPad Prism 9.0 software (GraphPad Software, La Jolla, CA, USA) was used for analysis and visualization of study results. *P* < 0.05 was considered to be statistically significant.

## Results

### Active ingredients and targets of SBG

According to the range of OB and DL, the 33 active components of SBG were obtained (Table 2), such as baicalein (MOL002714, OB = 33.52%, DL = 0.21), skullcapflavone II (MOL002927, OB = 69.51%, DL = 0.44), and moslosooflavone (MOL008206, OB = 44.09%, DL = 0.25). A total of 448 active components targets were obtained by combining the two databases. A network diagram (Figure S1**)** was generated and indicates that 33 active components and 448 drug targets had close relationships. In the component-target network diagram, the ellipses of different colors represent the targets of different components. Among them, baicalein (MOL002714) obtained more targets (Degree = 133) than other components, which were represented by a red ellipse.

**Table 2:** Information on the active components of *Scutellaria baicalensis* Georgi.

Molecular ID	CAS	Molecular name	Molecular formula	Molecular weight	OB(%)	DL	Degree
MOL002714	491-67-8	Baicalein	C_15_H_10_O_5_	270.25	33.52	0.21	133
MOL002934	55084-08-7	Skullcapflavone II	C_19_H_18_O_8_	374.37	69.51	0.44	121
MOL008206	3570-62-5	Moslosooflavone	C_17_H_14_O_5_	298.31	44.09	0.25	121
MOL000228	1090-65-9	(2R)-7-hydroxy-5-methoxy-2-phenylchroman-4-one (HMPO)	C_16_H_14_O_4_	270.30	55.23	0.20	120
MOL002915	19103-54-9	Salvigenin	C_18_H_16_O_6_	328.34	49.07	0.33	118
MOL002917	92519-93-2	Viscidulin II	C_17_H_14_O_7_	330.31	45.05	0.33	115
MOL002933	57096-02-3	8-Methoxyapigenin	C_16_H_12_O_6_	300.28	36.56	0.27	115
MOL002932	41060-16-6	Panicolin	C_17_H_14_O_6_	314.31	76.26	0.29	113
MOL000525	4443-09-8	Norwogonin	C_15_H_10_O_5_	270.25	39.40	0.21	111
MOL001490	117-81-7	Bis[(2S)-2-ethylhexyl] benzene-1,2-dicarboxylate (BEBD)	C_24_H_38_O_4_	390.62	43.59	0.35	109
MOL002908	77056-20-3	5,8,2′-Trihydroxy-7-methoxyflavone (TR7MF)	C_16_H_12_O_6_	300.28	37.01	0.27	104
MOL001689	480-44-4	Acacetin	C_16_H_12_O_5_	284.28	34.97	0.24	93
MOL002925	82475-00-1	5,7,2′,6′-Tetrahydroxyflavone (TEDF)	C_15_H_10_O_6_	286.25	37.01	0.24	82
MOL000358	83-46-5	Beta-sitosterol	C_29_H_50_O	414.79	36.91	0.75	79
MOL012246	57096-02-3	5,7,4′-Trihydroxy-8-methoxyflavanone (TR8MF)	C_16_H_14_O_6_	302.30	74.24	0.26	75
MOL002928	480-11-5	Oroxylin A	C_16_H_12_O_5_	284.28	41.37	0.23	74
MOL012245	94942-49-1	6-Methoxynaringenin	C_16_H_14_O_6_	302.30	36.63	0.27	74
MOL000449	83-48-7	Stigmasterol	C_29_H_48_O	412.77	43.83	0.76	70
MOL000173	632-85-9	Wogonin	C_16_H_12_O_5_	284.28	30.68	0.23	67
MOL000359	149-91-7	Sitosterol	C_29_H_50_O	414.79	36.91	0.75	47
MOL000552	86926-52-5	Tenaxin I	C_18_H_16_O_7_	344.34	31.71	0.35	33
MOL001458	3486-66-6	Coptisine	C_19_H_14_NO_4_^+^	320.34	30.67	0.86	33
MOL012266	70028-59-0	Rivularin	C_18_H_16_O_7_	344.34	37.94	0.37	31
MOL002937	18956-18-8	Dihydrooroxylin	C_16_H_14_O_5_	286.30	66.06	0.23	26
MOL002914	4049-38-1	Eriodyctiol (flavanone)	C_15_H_12_O_6_	288.27	41.35	0.24	25
MOL002897	1816598	Epiberberine	C_20_H_18_NO_4_^+^	336.39	43.09	0.78	22
MOL002910	479-54-9	Carthamidin	C_15_H_12_O_6_	288.27	41.15	0.24	19
MOL010415	56599-57-6	Methyl 11,13-eicosadienoate (MEA)	C_21_H_38_O_2_	322.59	39.28	0.23	19
MOL002909		5,7,2,5-Tetrahydroxy-8,6-dimethoxyflavone (TEDMF)	C_18_H_16_O_9_	376.34	33.82	0.45	13
MOL002879	25103-50-8	Diop	C_24_H_38_O_4_	390.62	43.59	0.39	8
MOL000073	35323-91-2	Ent-Epicatechin	C_15_H_14_O_6_	290.29	48.96	0.24	5
MOL001506	111-02-4	Supraene	C_30_H_50_	410.80	33.55	0.42	5
MOL002913	35683-17-1	Dihydrobaicalin_qt	C_15_H_12_O_5_	272.27	40.04	0.21	4

### Bone cancer pain targets and intersection targets

**Figure 2:**
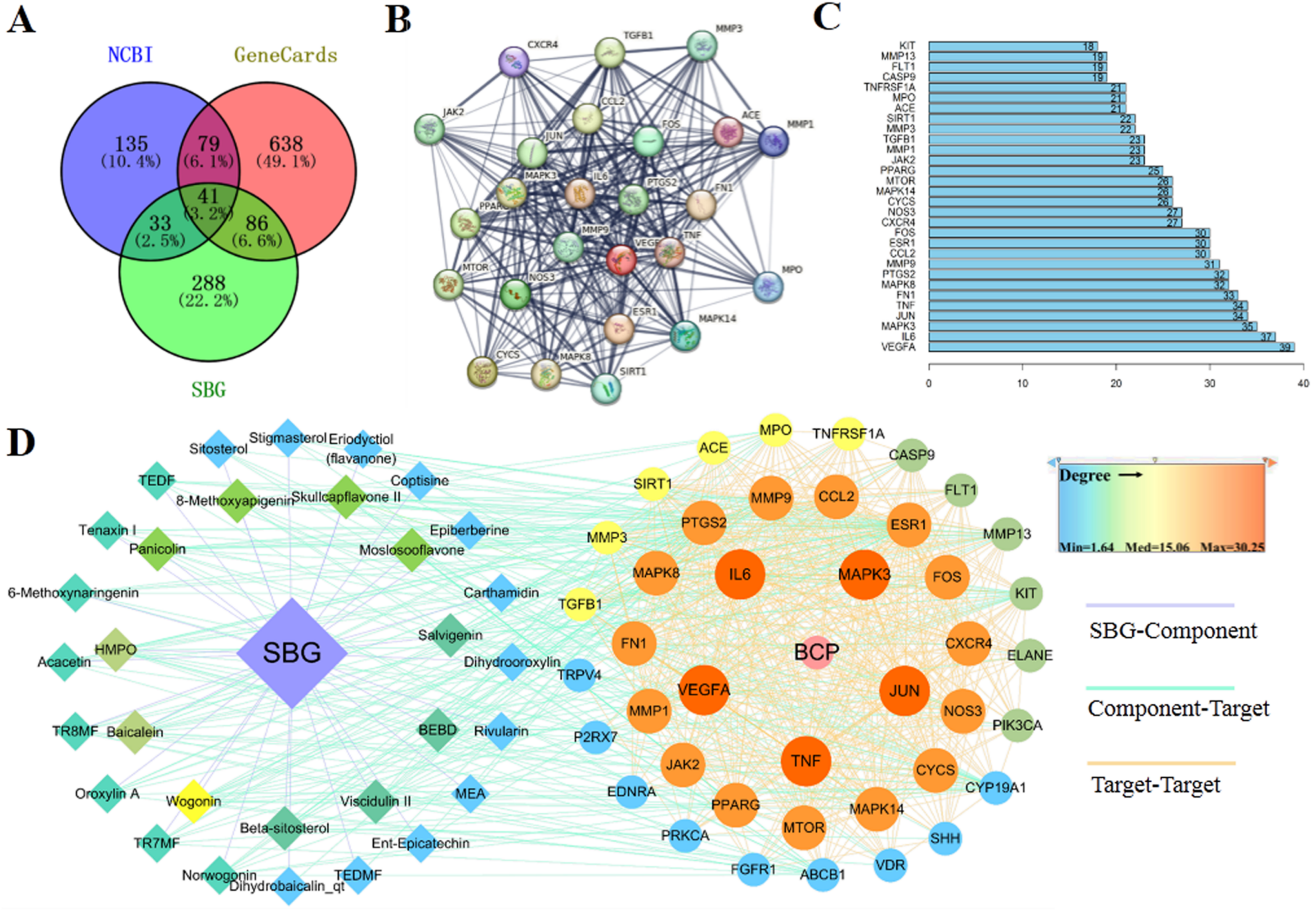
Diagrams of 41 Intersection targets for bone cancer pain and active components. (A) The Venn diagram. (B) The protein-protein interaction (PPI) network plotting. The lines of different colors represent different sources of the evidence for protein–protein interaction. (C) Number of connectivity degree of targets (Top 30). (D) Network of components and disease targets.

Based on the above results, 41 common targets were obtained through the intersection of SBG and BCP targets (Fig. 2A). The PPI diagram of 41 intersection targets was constructed (Fig. 2B), and degree value was counted (Fig. 2C). The targets in the center of the PPI diagram with degree value, and the closer connection with other potential targets, such as Vascular endothelial growth factor A (VEGFA), Interleukin-6 (IL6), MAP kinase ERK1 (MAPK3), Transcription factor AP-1 (JUN), and Tumor necrosis factor (TNF), which may be more essential in the treatment of BCP.

In the C-T-D network (Fig. 2D), targets with larger circle and closer to red may be more essential, such as VEGFA, MAPK3, and TNF. Meanwhile, the components with closer to the center of the circle and closer to yellow play an important role in the treatment of BCP, such as wogonin (MOL000173) and baicalein (MOL002714). In conclusion, the main components of SBG regulating BCP were baicalein and wogonin, and the core targets were VEGFA, IL6, MAPK3, JUN and TNF.

### GO and KEGG enrichment results

**Figure 3:**
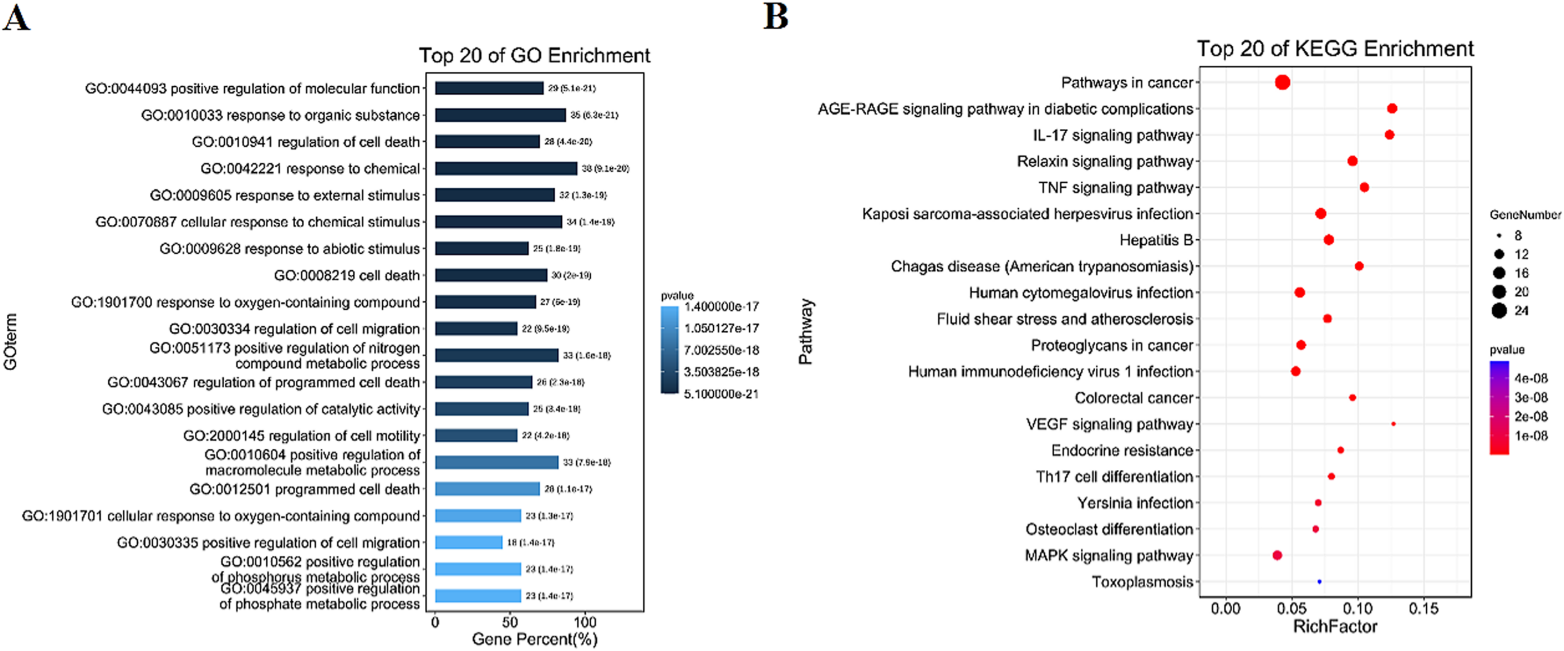
Diagrams of GO and KEGG enrichment analysis. (A) Barplot diagram of GO enrichment analysis (Top 20). (B) Bubble diagram of KEGG enrichment analysis (Top 20).

The intersection targets were analyzed by GO and KEGG enrichment, (Figs. 3A, 3B and S2). Intersection targets mainly regulated biological processes and related pathways, such as the regulation of cell death (GO:0010941), the regulation of cell migration (GO:0030334), the pathways in cancer (ko05200), IL-17 signaling pathway (ko04657), and TNF signaling pathway (ko04668). Results indicated that the active compounds of SBG alleviate BCP by regulating core targets of angiogenesis-related targets VEGF, Transforming growth factor beta-1 (TGFB1), Apoptosis-related targets Caspase-9 (CASP9), TNF, JUN, Estrogen receptor (ESR1), and MAPK3.

### Distribution of core targets in organs

**Figure 4:**
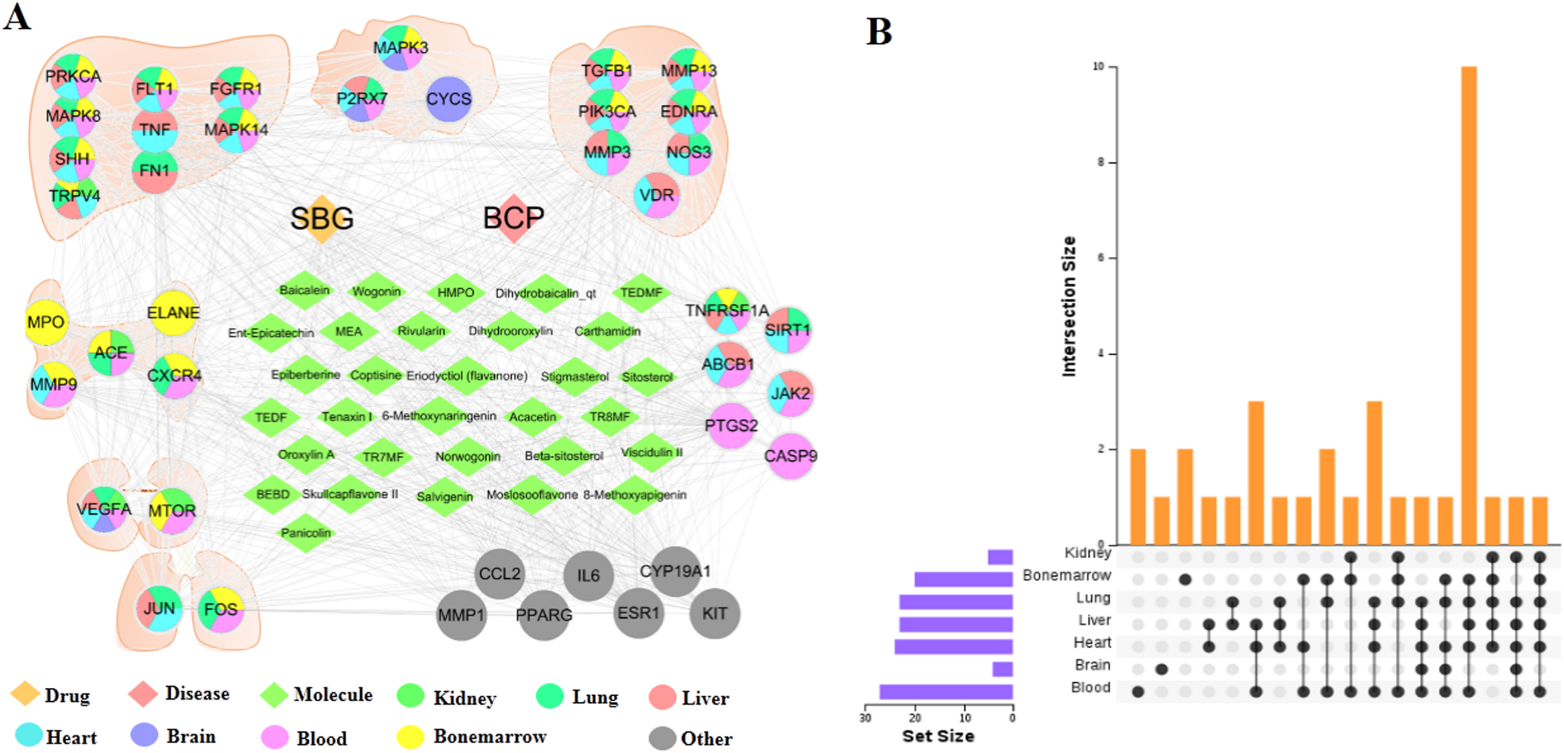
Distribution of intersection targets in major organs. (A) Network of components, disease targets and organs. (B) Venn diagram of intersection target distribution in major organs.

The tissue distribution of 41 intersection targets has shown in Figs. 4A and 4B. The results demonstrated that there were five targets (ACE, CXCR4, ELANE, MMP9, and MPO) mainly distributed in the bone marrow to regulate the proliferation, death, and apoptosis of bone cancer cells, and three targets (CYCS, MAPK3, and P2RX7) mainly distributed in the brain tissue may be related to the pain through regulation. In addition, there were six targets (ABCB1, CASP9, JAK2, PTGS2, SIRT1, and TNFRSF1A) mainly distributed in the blood, which may act on target organs by regulating the targets of the apoptosis, proliferation, and migration of the related cells in the blood. Most of the remaining targets were distributed in metabolic organs, such as liver, heart, and kidney.

### Cytotoxicity control test of drug

**Figure 5:**
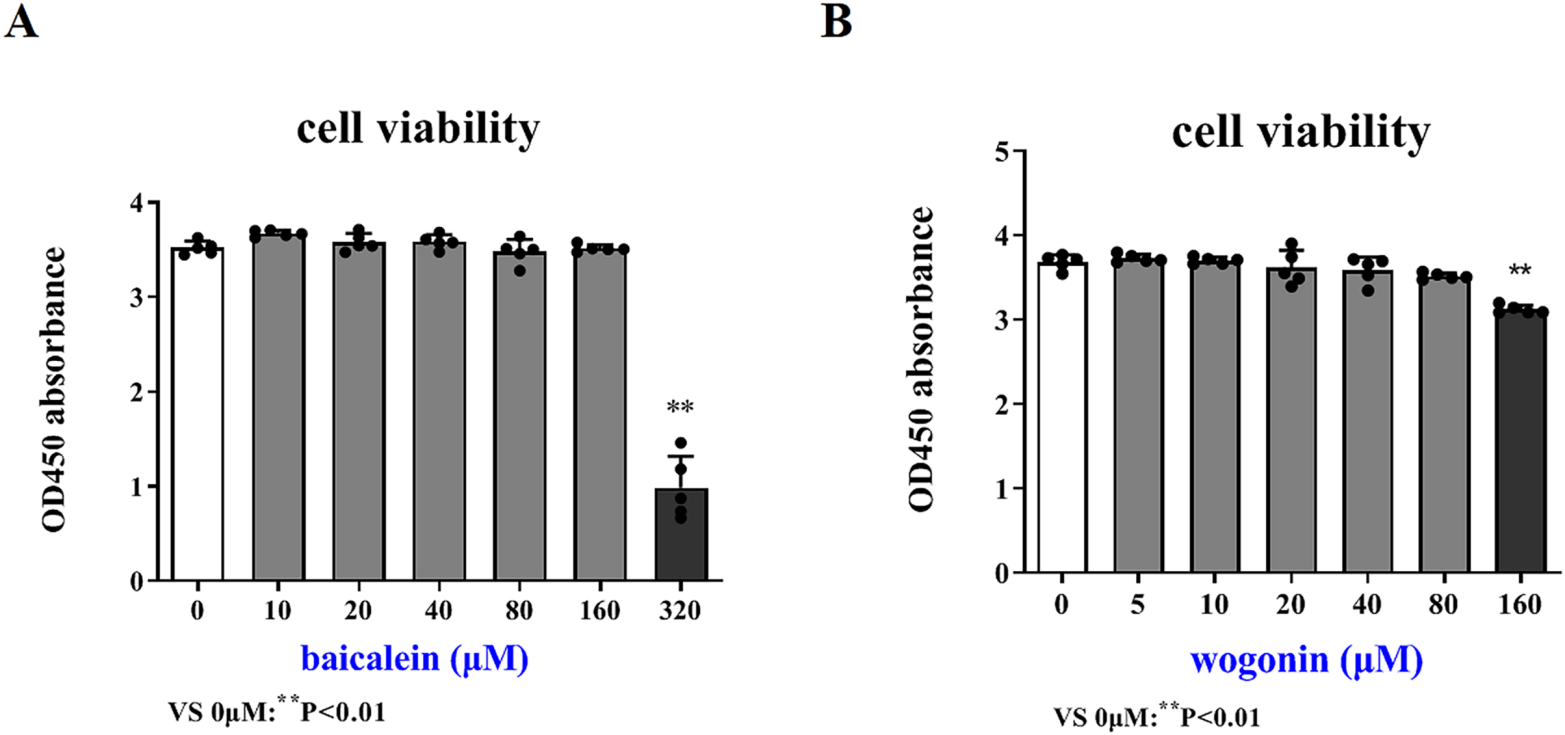
Safe drug concentrations of baicalein and wogonin in HT22 cells. Compared with 0 µM, **P < 0.01.

In order to more accurately examine the therapeutic effect of baicalein or wogonin on BCP, this study screened the safe concentration of baicalein or wogonin on the HT22 hippocampal neuron cell line (Fig. 5). The results demonstrated that baicalein did not affect the viability of HT22 cells in the concentration range of 0–160 μM, and *P* > 0.05. Similarly, wogonin did not affect the viability of HT22 cells in the concentration range of 0–80 μM (*P* > 0.05). Subsequent experiments will select 0–160 µM baicalein and 0–80 µM wogonin to explore the expression of core targets.

### Validation of key target results by *in vitro* assays

**Figure 6:**
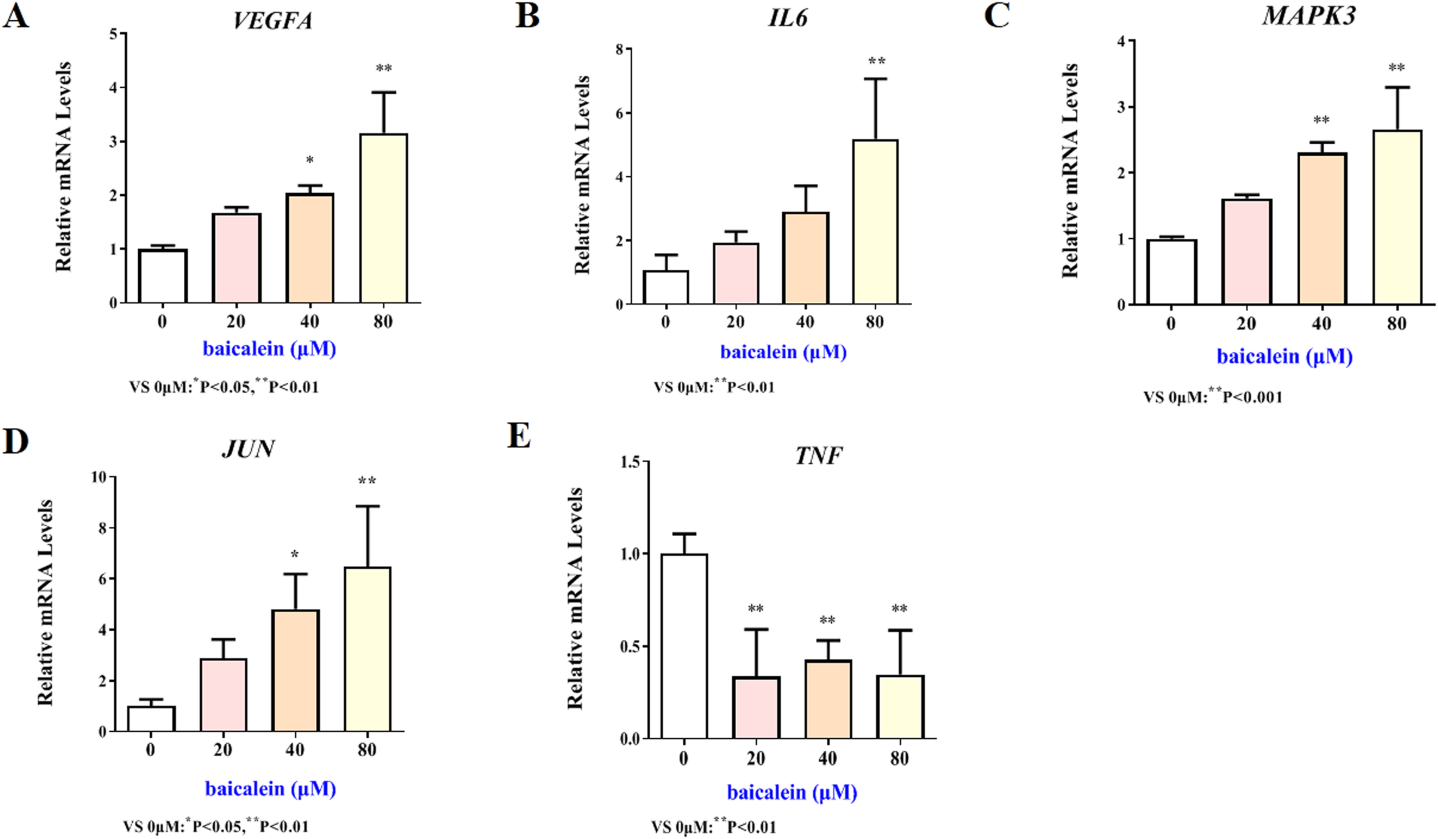
Expression of core targets after baicalein treatment. Compared with 0 µM, *P < 0.05, **P < 0.01.

**Figure 7:**
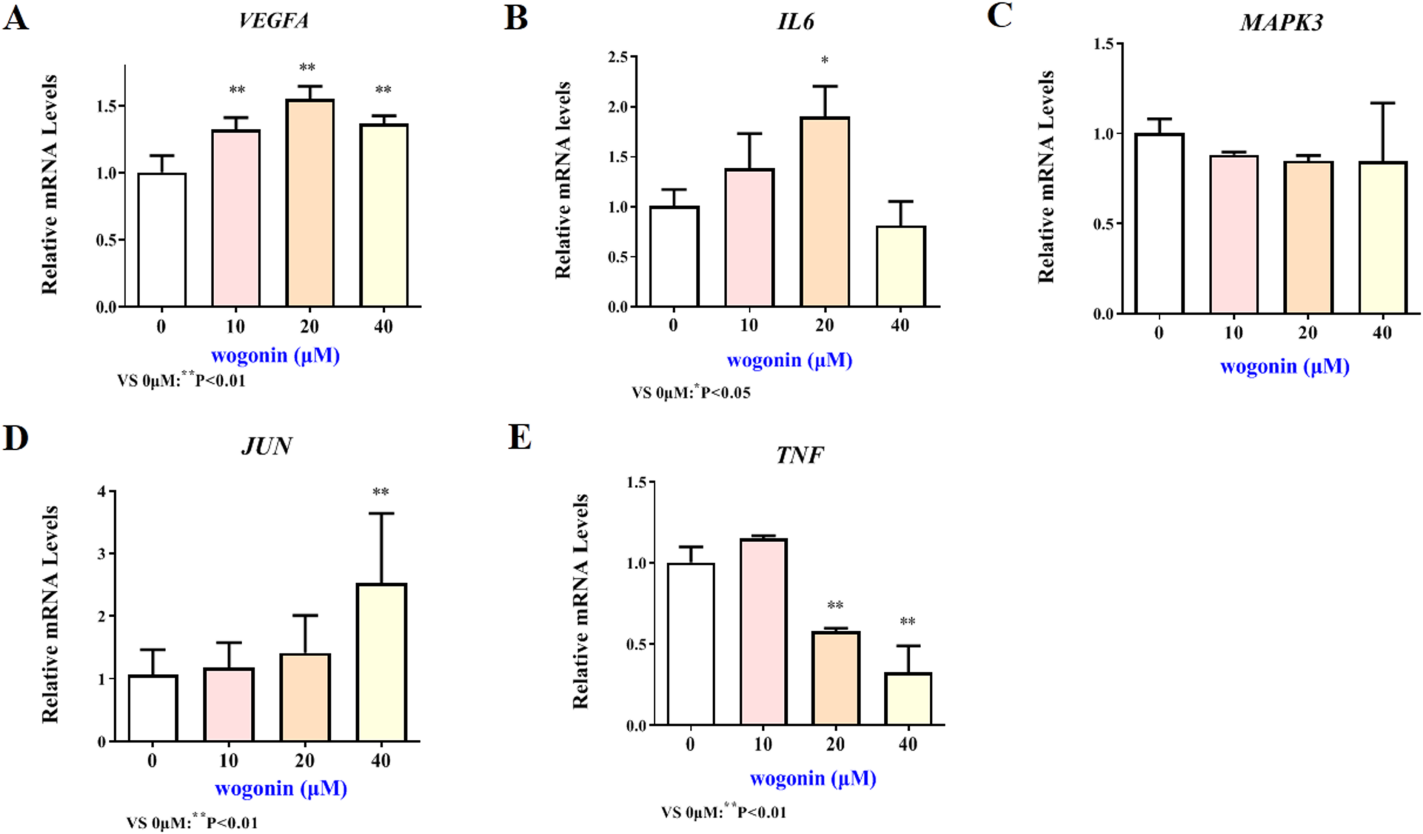
Expression of core targets after wogonin treatment. Compared with 0 µM, *P < 0.05, **P < 0.01.

The mRNA expression results of VEGFA, IL6, MAPK3, JUN and TNF target genes in HT22 cells after treatment of baicalein or wogonin were evidenced in Figs. 6 and 7. The results demonstrated that compared with the control group, baicalein obviously promoted the expression of VEGFA (40 and 80 µM), IL6 (80 µM), MAPK3 (40 and 80 µM), JUN (40 and 80 µM), and evidently reduced the expression of TNF (20, 40 and 80 µM). In addition, wogonin can significantly enhanced the expression of VEGFA (10, 20 and 40 µM), IL-6 (20 µM), JUN (40 µM), and obviously decreased the expression of TNF (20 and 40 µM), the difference has statistical reference value (*P* < 0.05). In general, the qRT-PCR results indicated that baicalein and wogonin were involved in hippocampal neurogenesis by regulating VEGFA, IL6, MAPK3, JUN and TNF target genes, thereby exerting anti-BCP effects.

## Discussion

Our research indicated that 33 active ingredients of SBG were related to 41 targets of BCP. The main flavonoids of SBG were baicalein, wogonin, oroxylin A, skullcapflavone II, and norwogonin. Pharmacokinetic studies had claimed that baicalin, baicalein, wogonin, wogonoside, and oroxylin A can be detected in rat plasma after administration, but baicalin and wogonoside absorption and elimination were quite slow in plasma (Tao et al., 2017; Cui et al., 2015), which is just as the prediction result of TCMSP showing baicalin (OB = 29.53%, DL = 0.77) and wogonoside (OB = 7.07%, DL = 0.77). Most glycoside components are hydrolyzed into aglycons by intestinal flora in the body, and they are difficult to reach target tissues or organs as prototype components. However, most drugs used to treat BCP are administered orally (Page, Mulvey & Bennett, 2020). In conclusion, compared with glycosides, the aglycones baicalein and wogonin in SBG may be more effective in the treatment of BCP.

Accumulating studies have illustrated that the active ingredients of SBG have a good activity and can exert anti-cancer effects by regulating cell apoptosis, death, metastasis, and autophagy. Baicalein can reduce the number of the differentiated osteoblasts by regulating the proliferation and apoptosis of preosteoblasts, impairing the angiogenesis of endothelial cells, and inhibiting the proliferation of synovial cells (Rasi Bonab et al., 2021). Baicalein has been studied to induce mitotic mutations, apoptosis, and autophagy through the NF-kB signaling pathway to inhibit the growth of human thyroid cancer cells (Yi et al., 2021). Wogonin inhibited the proliferation of ovarian cancer cells by activating apoptosis and cell cycle arrest (Liu et al., 2022), and it also increased tumor necrosis factor (TNF-α) and promoted the apoptosis of liver cancer cells (Zhao et al., 2019a). In this study, baicalein and wogonin were also screened as key components in the treatment of BCP. At the same time, experimental verification demonstrated that baicalein and wogonin could regulate the expression of VEGFA, IL6, MAPK3, JUN and TNF in HT22 hippocampal neurons. In short, SBG treatment of BCP mainly relies on baicalein and wogonin.

BCP involves peripheral, central, and glial-mediated nociceptive and neuropathic components (Wang et al., 2020). Neuropathic pain is an unpleasant sensory or emotional experience that induces morphological and functional changes in brain structure, especially in the plasticity of hippocampal neurons (Tyrtyshnaia et al., 2019). Restoration of impaired hippocampal neurogenesis helps reduce pain syndromes in various pain models (Gao & Shen, 2017). Currently, studies have found a reduction in the number of branches and dendritic lengths of hippocampal CA3 pyramidal neurons in mice with chronic sciatic constriction injury (CCI) (Tyrtyshnaia et al., 2019). Several clinical studies have demonstrated that a correlation between persistent pain and decreased hippocampal neurons (Zheng et al., 2017). Dysfunction of the prefrontal-hippocampal circuit has been identified as a major cause of pain and related working memory deficits (Cardoso-Cruz et al., 2019). In addition, studies have found that curcumin can alleviate CCI-induced neuropathic pain in rats by remarkably increasing the number of newborn neurons and improving hippocampal neurogenesis and synaptic plasticity (Du et al., 2021). Synaptoamine treatment ameliorated neuropathic pain in mice by reversing decreased dendritic density, worsening hippocampal neurogenesis, and long-term potentiation damage in ca1 pyramidal neurons (Tyrtyshnaia et al., 2021). In this study, the HT22 hippocampal neuron cell line was selected for exploration, and it was found that baicalein and wogonin could promote the occurrence, angiogenesis and proliferation of HT22 cells to relieve pain. In conclusion, baicalein and wogonin may play a role in alleviating BCP by improving the occurrence of hippocampal neurons.

At present, the pathological and therapeutic mechanisms of BCP have been reported. Chanling Gao exerted the analgesic effects in the cancer-induced bone pain (CIBP) rat model by inhibiting the IKKβ/NF-κB signaling pathway and the synthesis and release of TNF-α, IL-1β, and IL6 (Yang et al., 2020). Aitongxiao reduced the level of IL-1β, TNF-α, and prostaglandin E2 (PGE2) to alleviate the painful symptoms of tibia cancer pain in rats (Mao et al., 2021). The inhibition of endoplasmic reticulum stress triggered by inflammatory mediators (TNF-α, IL-1β, and IL-6) in spinal cord neurons can alleviate BCP by regulating neuroinflammation (Mao et al., 2020). Most of the studies on the mechanism of BCP focus on the regulation of inflammation, and this study found that hippocampal neurons also play an essential role. Ketamine can relieve pain-induced depressive symptoms by enhancing the release of VEGFA and promoting neurogenesis in the hippocampus (Deyama & Duman, 2020). As a multi-effect cytokine, IL-6 may promote the survival of hippocampal neurons to relieve pain (Liao et al., 2022; Del Giudice & Gangestad, 2018). Cannabinoids can also enhance the expression of MAPK3 in HT22 hippocampal cells to protect nerve pain relief (Olianas, Dedoni & Onali, 2021). Furthermore, it has been found that reduction of TNF levels significantly ameliorate CCI-induced neuropathic pain in mice, and this behavioral effect is associated with changes in hippocampal neurogenesis and plasticity (Grilli, 2017). In this study, baicalein and wogonin can also activate the expression of VEGFA, IL6, MAPK3 and JUN and reduce the expression of TNF, which alleviated BCP by regulating the occurrence of hippocampal neurons. Taken together, the mechanism of baicalein and wogonin alleviating BCP may be related to their promotion of hippocampal neurogenesis.

Recently, baicalein and wogonin have been reported in the literature on pathway regulation. Studies have found that baicalein can target TLR4/HIF-1α/VEGF signaling pathway in the treatment of colorectal cancer (Chen et al., 2021). Baicalein induced apoptosis and autophagy in breast cancer cells by inhibiting PI3K/AKT pathway (Yan et al., 2018), and wogonin also alleviated renal tubular epithelial damage in diabetic nephropathy by inhibiting PI3K/AKT pathway (Lei et al., 2021). Baicalein and wogonin also regulated the MAPK pathway to reduce amyloid-induced toxicity and exert neuroprotective effects (Ji et al., 2020). Wogonin promoted hematoma clearance and improves neurological function in a mouse model of intracerebral hemorrhage by modulating the PPAR-γ pathway (Zhuang et al., 2021). Additionally, wogonin can also regulated the IL-17 signaling pathway to improve obesity-induced lipid metabolism disorder and cardiac injury (Zhou & Yin, 2022). In this study, the pathways of SBG treatment of BCP were mainly enriched in pathways in cancer, which involved VEGF, PI3K/AKT, MAPK and PPAR signaling pathways. Among the top 20 pathways, SBG was also involved in the regulation of IL-17 and TNF signaling pathways. In a nutshell, baicalein and wogonin, the main components of SBG, can regulate VEGF, PI3K/AKT, MAPK, PPAR and IL-17 signaling pathways and participate in the occurrence and development of tumors or neurological diseases.

In summary, this study only predicted and preliminarily validated the mechanism of SBG in treating BCP. Although research has discovered key targets and mechanisms for regulating and treating BCP, a large number of *in vivo* and *in vitro* verification experiments are still needed. Only in this way, we can fully reveal the mechanism of SBG in treating BCP and provide a theoretical basis for its targeted therapy.

## Conclusion

In conclusion, the network pharmacology of this study was used to screen out the active compounds of SBG (baicalein and wogonin), 41 intersection targets (VEGFA, IL6, MAPK3, JUN, TNF, and other targets), and 20 main pathways (Pathways in cancer, IL-17, TNF signaling pathway and others). Finally, experiments *in vitro* were used to verify that baicalein and wogonin regulate the expression of key targets (VEGFA, IL6, MAPK3, JUN, and TNF) in HT22 hippocampal cells. This study provides new ideas and theoretical basis for the mechanism of action of SBG in the treatment of BCP.

## Supplemental Information

10.7717/peerj.14394/supp-1 Supplemental Information 1 SBG composition and target network diagram.

10.7717/peerj.14394/supp-2 Supplemental Information 2 Pathways in cancer.

10.7717/peerj.14394/supp-3 Supplemental Information 3 Raw Data.

## Additional Information and Declarations

### Data Availability

The following information was supplied regarding data availability: The raw data are available in the Supplemental Files.
