# Supplementary figures and images for: Regulatory mechanism of Scutellaria baicalensis Georgi on bone cancer pain based on network pharmacology and experimental verification
Source: PeerJ. 2022 Nov 17;10:e14394. doi: 10.7717/peerj.14394 (PMC9676018; doi:10.7717/peerj.14394)

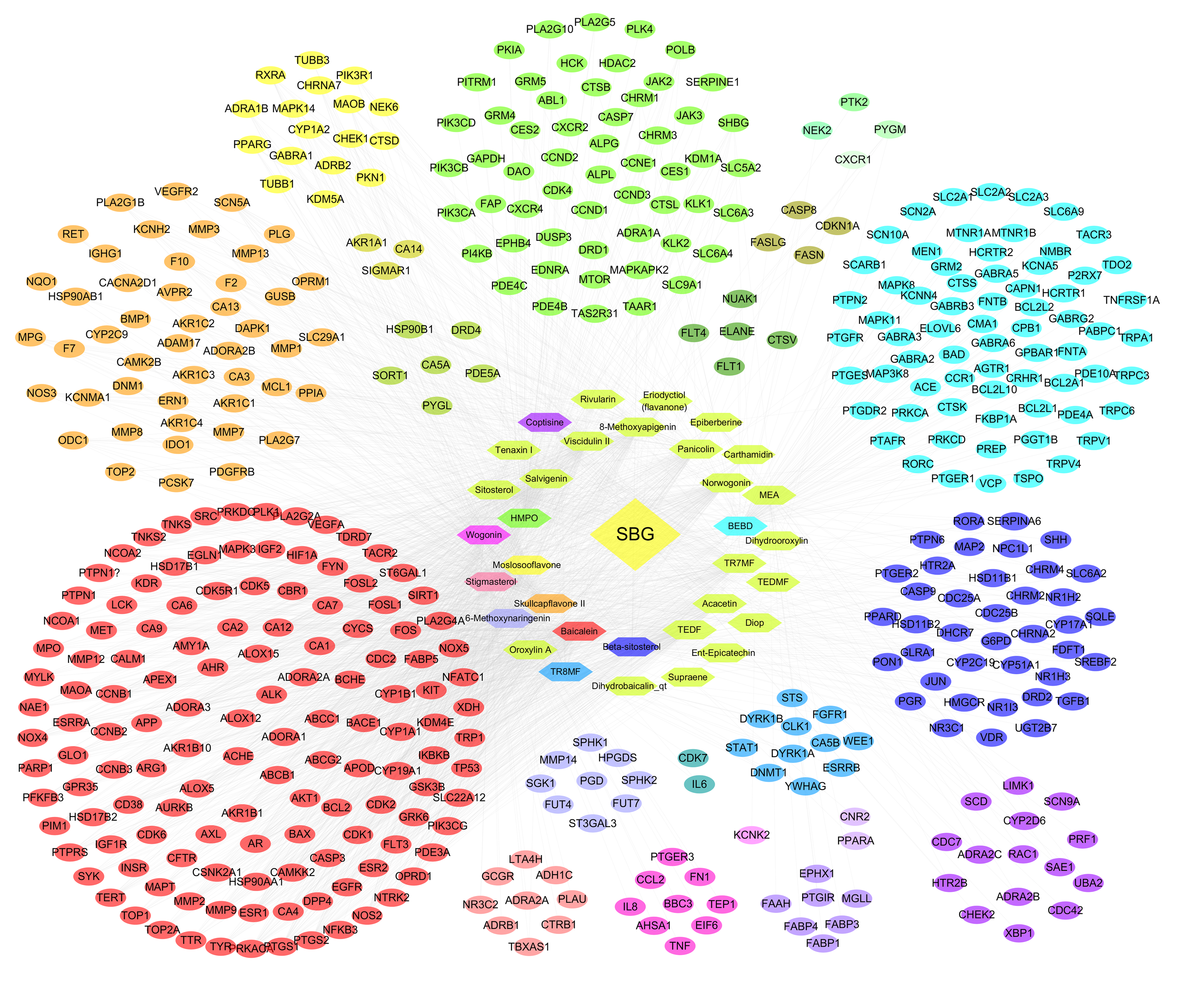

Supplement: Supplemental Information 1 [file peerj-10-14394-s001.png]

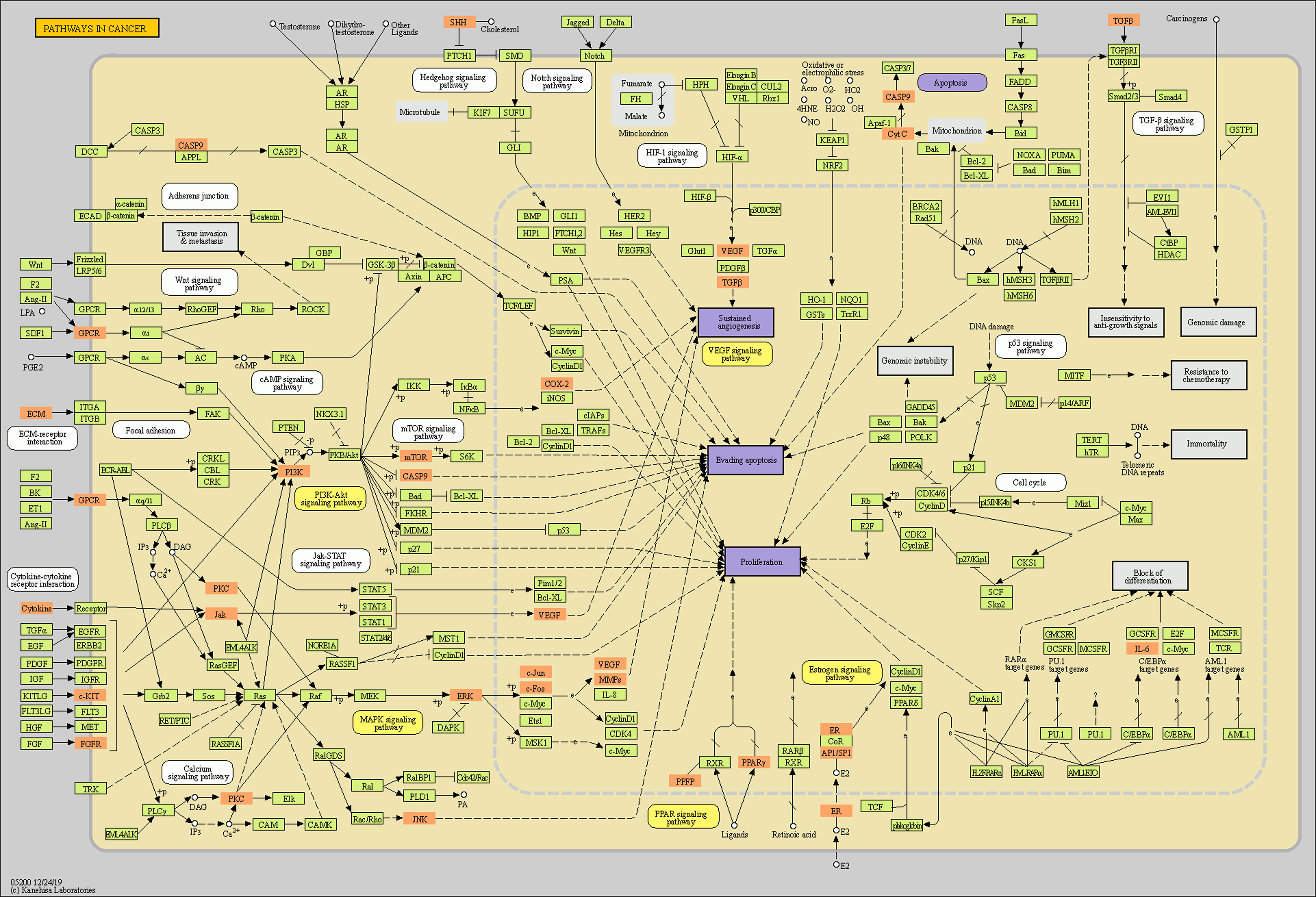

Supplement: Supplemental Information 2 [file peerj-10-14394-s002.png]
